# Association between estimated pulse wave velocity and in-hospital mortality of patients with acute kidney injury: a retrospective cohort analysis of the MIMIC-IV database

**DOI:** 10.1080/0886022X.2024.2313172

**Published:** 2024-02-15

**Authors:** Xinhai Cui, Yuanlong Hu, Dongxiao Li, Mengkai Lu, Zhiyuan Zhang, Dongfang Kan, Chao Li

**Affiliations:** a College of Traditional Chinese Medicine, Shandong University of Traditional Chinese Medicine, Jinan, China; b First Clinical Medical College, Shandong University of Traditional Chinese Medicine, Jinan, China; c Innovation Research Institute of Traditional Chinese Medicine, Shandong University of Traditional Chinese Medicine, Jinan, China

**Keywords:** Acute kidney injury, estimated pulse wave velocity, in-hospital mortality, MIMIC-IV database

## Abstract

**Background:**

Estimated pulse wave velocity (ePWV) has been found to be an independent predictor of cardiovascular mortality and kidney injury, which can be estimated noninvasively. This study aimed to investigate the association between ePWV and in-hospital mortality in critically ill patients with acute kidney injury (AKI).

**Methods:**

This study included 5960 patients with AKI from the Medical Information Mart for Intensive Care IV (MIMIC-IV) database. The low and high ePWV groups were compared using a Kaplan-Meier survival curve to evaluate the differences in survival status. Cox proportional hazards models were used to explore the association between ePWV and in-hospital mortality in critically ill patients with AKI. To further examine the dose-response relationship, we used a restricted cubic spline (RCS) model. Stratification analyses were conducted to investigate the effect of ePWV on hospital mortality across various subgroups.

**Results:**

Survival analysis indicated that patients with high ePWV had a lower survival rate than those with low ePWV. Following adjustment, high ePWV demonstrated a statistically significant association with an increased risk of in-hospital mortality among AKI patients (HR = 1.53, 95% CI = 1.36-1.71, *p* < 0.001). Analysis using the RCS model confirmed a linear increase in the risk of hospital mortality as the ePWV values increased (*P* for nonlinearity = 0.602).

**Conclusions:**

A high ePWV was significantly associated with an increased risk of in-hospital mortality among patients with AKI. Furthermore, ePWV was an independent predictor of in-hospital mortality in critically ill patients with AKI.

## Introduction

Acute kidney injury (AKI) is characterized by a decline in kidney function, typically involving an abrupt decrease in glomerular filtration rate, resulting in nitrogenous waste product accumulation and an inability to maintain fluid and electrolyte homeostasis [1]. AKI is a serious complication among critically ill hospital patients, with an incidence of up to 24% among trauma patients admitted to the intensive care unit (ICU), and is associated with high morbidity and mortality rates [2,3]. Despite efforts to pursue advances in care, the mortality rate for patients requiring kidney replacement therapy remains approximately 50% [4]. Consequently, the prevention and early detection of AKI must be prioritized, and exploring factors related to death in patients with AKI is necessary.

Arterial stiffness can indicate decline in kidney function [5]. Higher arterial stiffness increases flow and pressure pulsatility, inducing premature recovery of systolic reflex waves, and eventually reducing the damping function of the arteries [5]. In particular, pulsating blood flow travels to the hardened aorta and extends to organs with high blood flow and low microvascular resistance, such as the brain and kidneys, inducing damage to the glomeruli [6]. Pulse wave velocity (PWV) describes the speed of pulse pressure traveling along the length of the artery, measured in meters per second, and is calculated by dividing the distance between the carotid and femoral arteries by time [7]. PWV is considered the gold standard for arterial stiffness measurement in clinical settings and is associated with an increased risk of cardiovascular morbidity and mortality [8,9]. Additionally, previous research demonstrated that a one-unit increase in PWV score was significantly associated with a 1.5 fold greater risk of cardiac surgery-associated AKI events (*p* = 0.006, 95% CI = 1.13-2.10) [10]. PWV was also an independent predictor of postoperative AKI following off-pump coronary artery bypass grafting in 164 patients [11]. These findings indicate that PWV is a recognized risk factor for AKI development after surgery.

PWV can be estimated using chronological age and blood pressure as inputs through the use of estimated pulse wave velocity (ePWV), a non-measurement technique for assessing arterial stiffness that was recently developed [12]. Evidence indicates that the difference between ePWV and carotid femoral pulse wave velocity (cfPWV) is relatively small, with a relative difference of −0.3% (95% CI = −15-17%). Furthermore, ePWV was found to predict major cardiovascular events independently from systematic coronary risk evaluation, Framingham risk score, and cfPWV [13]. Similar to PWV, ePWV can also predict AKI and mortality following colorectal surgery [14]. However, research on the relationship between ePWV and AKI has focused more on surgical patients. This exploratory study aimed to investigate the association between ePWV and in-hospital mortality among critically ill patients with AKI, which could yield a fast and reliable method for identifying the risk of AKI.

## Methods

### Study population

**Figure 1.**
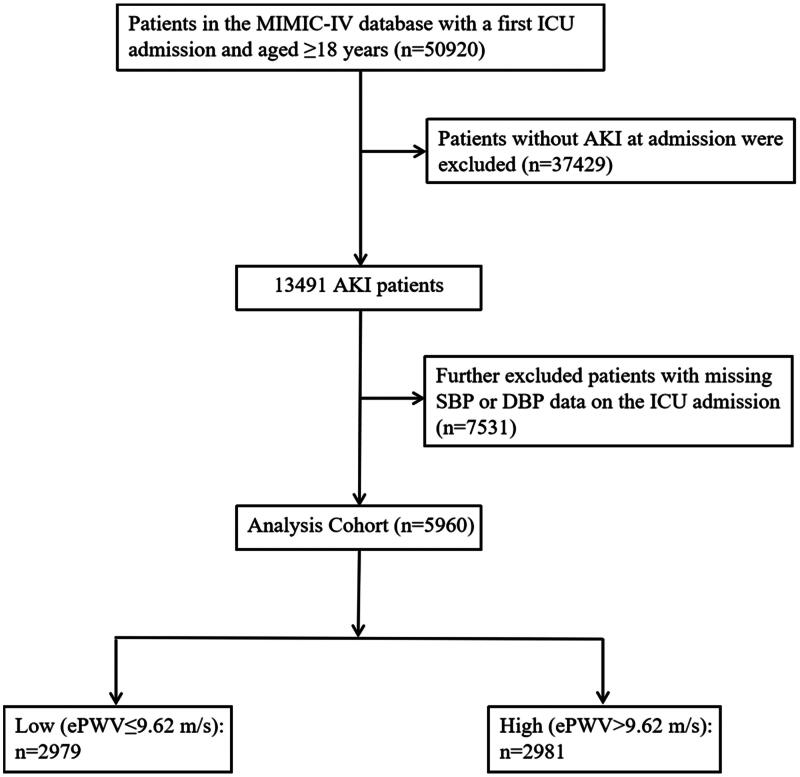
The flowchart of patients’ selection.

The data in this study were obtained from the publicly accessible Medical Information Mart for Intensive Care IV (MIMIC-IV) database, which comprises more than 40,000 ICU inpatients at the Beth Israel Deaconess Medical Center in Boston, between 2008 and 2019. Xinhai Cui, one of the authors of this paper, registered for and completed the courses to obtain permission to use the dataset. Patients with AKI meeting Kidney Disease Improving Global Outcomes (KDIGO) creatinine criteria were included, in accordance with the International Classification of Diseases (ICD-9) diagnostic code (5845-5849) of the database. The following were considered the inclusion criteria: AKI diagnosed as specifically evidenced by an increase in serum creatinine levels by ≥ 0.3 mg/dL (≥ 26.5 μmol/L) within 48 h, or an increase in serum creatinine to ≥ 1.5 times baseline within the previous 7 days, or a urine volume < 0.5 mL/kg/h for 6 h [15]. A patient was eliminated if they were not first ICU admission, were under the age of 18, lacked patient’s blood pressure information such as systolic blood pressure (SBP) and diastolic blood pressure (DBP). Eventually, the study cohort included 5960 patients, divided into two groups according to their ePWV medians on ICU admission (Figure 1).

### Data collection

The patients’ data was obtained from the MIMIC-IV database: (1) clinical parameters: age, gender, race, SBP, DBP, ePWV, temperature, pulse oxime-try-derived oxygen saturation (SpO_2_), simplified acute physiological score II (SAPSII), systemic inflammatory response syndrome (SIRS) score, acute physiology score III (APSIII), and acute kidney injury network (AKIN); (2) laboratory parameters: white blood cell (WBC), lymphocyte, neutrophil, platelet, hemoglobin, serum creatinine (Scr), blood urea nitrogen (BUN), albumin, glucose, serum potassium, serum sodium, blood pH, total bilirubin, and bicarbonate; (3) comorbidities: heart failure, hypertension, coagulopathy, asystole, respiratory failure, sepsis, diabetes, chronic kidney disease (CKD), liver diseases, atrial fibrillation (AF); (4) medication use: vasopressin; (5) procedures: renal replacement therapy (RRT); (6) events: los hospital and mortality. Only the data recorded during the initial ICU admission measurements were used for analysis.

### Assessment of ePWV and outcome

Mean arterial pressure (MAP) was calculated as (DBP) + 0.4 × (SBP-DBP) [16]. The ePWV was determined based on the following equation [12], which considers age and MAP: ePWV = 9.587 − 0.402 × age + 4.560 × 10^−2^ × age^2^ - 2.621 × 10^−5^ × age^2^ × MAP + 3.176 × 10^−3^ × age × MAP − 1.832 × 10^−2^ × MAP. In this study, the primary endpoint was in-hospital mortality in patients with AKI admitted to the ICU.

### Statistical analysis

Baseline patient characteristics were presented as means and standard deviations for continuous variables, while frequencies and percentages were used for categorical variables. Differences between baseline characteristics were analyzed using either the t-test or the Mann-Whitney U test, depending on the distribution of the data. The missForest R package was used to fill any gaps in the data by utilizing the random forests strategy, which helps with missing value imputation. We utilized Kaplan-Meier survival curves to evaluate survival rates in both the low and high ePWV groups, with differences among the groups assessed using log-rank tests. Cox proportional hazards models (Cox R function) were used to determine the independent association between ePWV and in-hospital mortality among patients with AKI. In non-adjusted model, no covariates were adjusted; in Model 1, adjustments were made for gender, whereas Model 2 additionally adjusted for temperature, SpO2, SAPSII, SIRS score, APSIII, WBC, lymphocyte, neutrophil, platelet, hemoglobin, Scr, BUN, albumin, glucose, serum potassium, serum sodium, blood pH, total bilirubin, bicarbonate, heart failure, hypertension, coagulopathy, asystole, respiratory failure, sepsis, diabetes, CKD, liver diseases, AF, use of vasopressors and RRT. A restricted cubic spline (RCS) model was implemented to explore whether a dose-response relationship exists between ePWV and in-hospital mortality in patients with AKI. A likelihood ratio test was conducted to detect potential nonlinearity. Finally, the analyses were further stratified by age (≤ 65 years and > 65 years), gender (female and male), heart failure, hypertension, coagulopathy, asystole, respiratory failure, sepsis, diabetes, CKD, liver diseases, AF, vasopressin, and RRT to assess whether the effect of ePWV on in-hospital mortality differed across subgroups. Interactions between ePWV and the variables used in stratification were examined using likelihood ratio tests. All analyses were performed using Stata/MP software (version 16.0; StataCorp LLC, College Station, TX, USA) and R (version 4.3.2; R Foundation for Statistical Computing, Vienna, Austria) with RStudio (version 2023.03.0 + 386; PBC, Boston, MA, USA).

## Results

### Baseline characteristics of included patients

The study ultimately enrolled 5960 patients, as shown in Figure 1. Baseline patient characteristics were compared between the low ePWV group (ePWV ≤ 9.62 m/s, 2979 participants) and the high ePWV group (ePWV > 9.62 m/s, 2981 participants) (Table 1). The mean levels of ePWV of the two groups were 7.57 ± 1.34 m/s and 11.98 ± 1.82 m/s, respectively. Patients with high ePWV were generally older, female, higher levels of neutrophil, platelet, BUN, albumin, serum podium, blood pH and bicarbonate, higher prevalence of heart failure, hypertension, diabetes, AF compared to the low group. With increasing ePWV, hospital length of stay decreased gradually (17.99 days vs. 14.98 days), while hospital mortality increased (26.25% vs. 27.88%).

**Table 1.** Baseline characteristics of critical ill patients with AKI grouped according to ePWV.

Characteristic	Overall (*n* = 5960)	Low (*n* = 2979)	High (*n* = 2981)	P-value
Clinical parameters				
Age, n%				< 0.001
≤ 65	2696 (45.23)	2370 (79.56)	326 (10.94)	
> 65	3264 (54.77)	609 (20.44)	2655 (89.06)	
Gender, n%				< 0.001
Female	2230 (37.42)	1008 (33.84)	1222 (40.99)	
Male	3730 (62.58)	1971 (66.16)	1759 (59.01)	
Race, n%				0.800
White	4252 (71.34)	2130 (71.50)	2122 (71.18)	
Black	488 (8.19)	250 (8.39)	238 (7.98)	
Other	1220 (20.47)	599 (20.11)	621 (20.83)	
SBP, mmHg	117.88 (29.51)	108.02 (22.96)	127.72 (31.94)	< 0.001
DBP, mmHg	58.71 (17.17)	56.25 (13.68)	61.16 (19.75)	< 0.001
ePWV, m/s	9.78 (2.72)	7.57 (1.34)	11.98 (1.82)	< 0.001
Temperature, °C	36.21 (3.75)	35.91 (5.20)	36.51 (0.96)	< 0.001
SpO_2_, %	64.37 (27.08)	65.00 (27.37)	63.65 (26.73)	0.072
SAPSII	46.30 (15.68)	44.10 (16.25)	48.51 (14.77)	< 0.001
SIRS score	2.87 (0.91)	2.94 (0.90)	2.84 (0.92)	< 0.001
APSIII	61.03 (25.08)	63.24 (26.57)	58.83 (23.30)	< 0.001
AKIN, n%				0.078
I	14 (0.23)	10 (0.33)	4 (0.13)	
II	84 (1.41)	49 (1.64)	35 (1.17)	
III	5862 (98.36)	2920 (98.02)	2942 (98.69)	
Laboratory parameters				
WBC, K/uL	14.05 (10.47)	14.33 (9.66)	13.76 (11.22)	< 0.001
Lymphocyte, %	11.84 (9.22)	12.06 (9.77)	11.62 (8.63)	0.800
Neutrophil, %	78.16 (12.24)	77.56 (12.80)	78.77 (11.63)	0.002
Platelet, K/uL	202.96 (106.49)	198.80 (111.30)	207.12 (101.31)	< 0.001
Hemoglobin, g/dL	10.38 (2.33)	10.40 (2.47)	10.36 (2.19)	0.500
Scr, mg/dL	1.95 (1.54)	2.10 (1.78)	1.80 (1.25)	< 0.001
BUN, mg/dL	37.36 (25.91)	36.79 (26.98)	37.94 (24.79)	< 0.001
Albumin, g/dL	3.00 (0.64)	2.97 (0.70)	3.03 (0.59)	< 0.001
Glucose, mg/dL	159.70 (88.25)	161.32 (97.23)	158.08 (78.23)	0.046
Serum potassium, Eq /L	4.42 (0.89)	4.44 (0.93)	4.41 (0.84)	> 0.900
Serum podium, mEq/L	137.43 (5.79)	136.93 (5.94)	137.92 (5.59)	< 0.001
Blood pH	7.33 (0.12)	7.32 (0.13)	7.35 (0.11)	< 0.001
Total bilirubin, mg/dL	2.02 (4.50)	2.71 (5.71)	1.34 (2.64)	< 0.001
Bicarbonate, mmol/L	21.79 (4.13)	21.53 (4.12)	22.05 (4.13)	< 0.001
Comorbidities				
Heart failure, n%	2515 (42.20)	1023(34.34)	1492 (50.05)	< 0.001
Hypertension, n%	2134 (35.81)	1020 (34.24)	1114 (37.37)	0.012
Coagulopathy, n%	909 (15.25)	501 (16.82)	408 (13.69)	< 0.001
Asystole, n%	309 (5.18)	139 (4.67)	170 (5.70)	0.071
Respiratory failure, n%	3378 (56.68)	1757 (58.98)	1621 (54.38)	< 0.001
Sepsis, n%	1683 (28.24)	905 (30.38)	778 (26.10)	< 0.001
Diabetes, n%	2302 (38.62)	1108 (37.19)	1194 (40.05)	0.023
CKD, n%				0.130
I	9 (0.15)	4 (0.13)	5 (0.17)	
II	100 (1.68)	48 (1.61)	52 (1.74)	
III	503 (8.44)	209 (7.02)	294 (9.86)	
IV	142 (2.38)	56 (1.88)	86 (2.88)	
V	20 (0.34)	12 (0.40)	8 (0.27)	
Unknown	778 (13.05)	292 (9.80)	486 (16.30)	
Liver diseases, n%	301 (5.05)	223 (7.49)	78 (2.62)	< 0.001
AF, n%	2581 (43.31)	923 (30.98)	1658 (55.62)	< 0.001
Medication use				
Vasopressin, n%	2124 (35.64)	1237 (41.52)	887 (29.76)	< 0.001
Procedures				
RRT, n%	779 (13.07)	481 (16.15)	298 (10.00)	< 0.001
Events				
Los Hospital, days	16.49 (15.73)	17.99 (17.53)	14.98 (13.54)	< 0.001
Hospital mortality, n%	1613 (27.06)	782 (26.25)	831 (27.88)	0.200

SBP, systolic blood pressure; DBP, diastolic blood pressure; SpO_2_, pulse oxime-try-derived oxygen saturation; SAPSII, Simplified Acute Physiology Score II; SIRS, systemic inflammatory response syndrome score; APSIII, Acute Physiology Score III; AKIN, Acute Kidney Injury Network; WBC, white blood cell; Scr, serum creatinine; BUN, blood urea nitrogen; CKD, chronic kidney disease; AF, atrial fibrillation; RRT, renal replacement therapy.

### Relationship between ePWV and in-hospital mortality of patients with AKI

**Figure 2.**
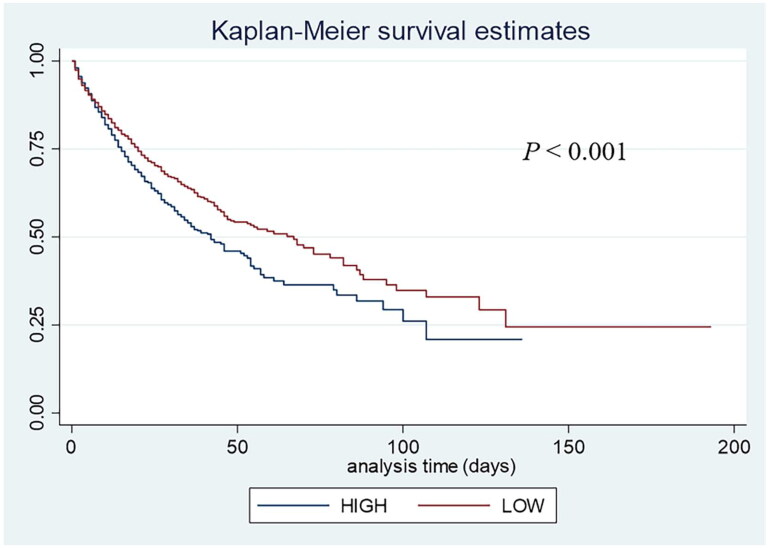
Kaplan-Meier survival curves for in-hospital mortality among low ePWV group and high ePWV group.

**Figure 3.**
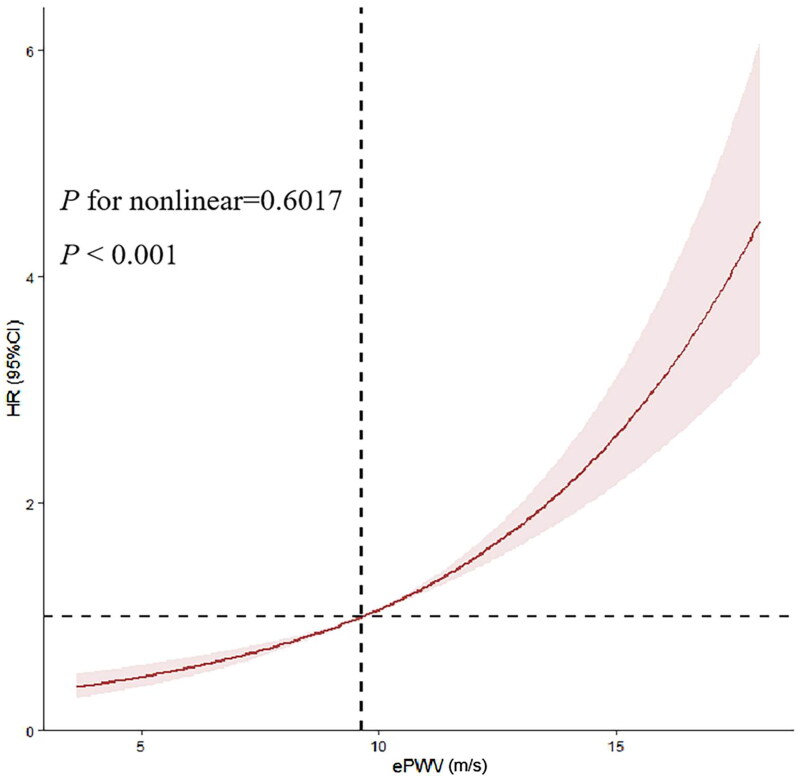
RCS curve for the ePWV hazard ratio and hospital mortality.

Based on the results, out of the 5960 patients, there were 1613 in-hospital deaths, accounting for 27.06% of the total. The Kaplan-Meier survival curves indicated a progressively worse survival probability for AKI patients as the ePWV levels increased (*p* < 0.001) (Figure 2). Moreover, significant intergroup differences in mortality rates during hospitalization were evident (log-rank *p* < 0.001). As shown in Table 2, each increase of one m/s in ePWV was associated with a 22% increased risk of death among patients with AKI (HR = 1.22, 95% CI = 1.11-1.35, *p* < 0.001). This positive association persisted after adjusting for gender in model 1 (HR = 1.20, 95% CI = 1.09-1.33, *p* < 0.001) and even after adjusting for all covariates in model 2 (HR = 1.53, 95% CI =1.36-1.71, *p* < 0.001). Notably, the RCS model revealed a linear increase in the risk of in-hospital mortality with increasing ePWV (*P* for non-linearity = 0.602) (Figure 3). It is suggested that when ePWV is larger than 9.65 m/s, there is a significant association with an increased risk of in-hospital mortality.

**Table 2.** Cox proportional HR for in-hospital mortality.

	Non-adjusted Model		Model 1		Model 2	
Categories	HR (95% CI)	P-value	HR (95% CI)	P-value	HR (95% CI)	P-value
Low (*n* = 2979)	Ref.		Ref.		Ref.	
High (*n* = 2981)	1.22 (1.11-1.35)	<0.001	1.20 (1.09-1.33)	<0.001	1.53 (1.36-1.71)	<0.001

Non-adjusted Model: unadjusted.

Model 1: adjusted for gender.

Model 2: adjusted for gender, temperature, SpO_2,_ SAPSII, SIRS score, APSIII, WBC, lymphocytes, neutrophils, platelets, hemoglobin, Scr, BUN, albumin, glucose, serum potassium, serum sodium, blood pH, total bilirubin, bicarbonate, heart failure, hypertension, coagulopathy, asystole, respiratory failure, sepsis, diabetes, CKD, liver diseases, AF, vasopressin, and RRT.

### Subgroup analysis

**Figure 4.**
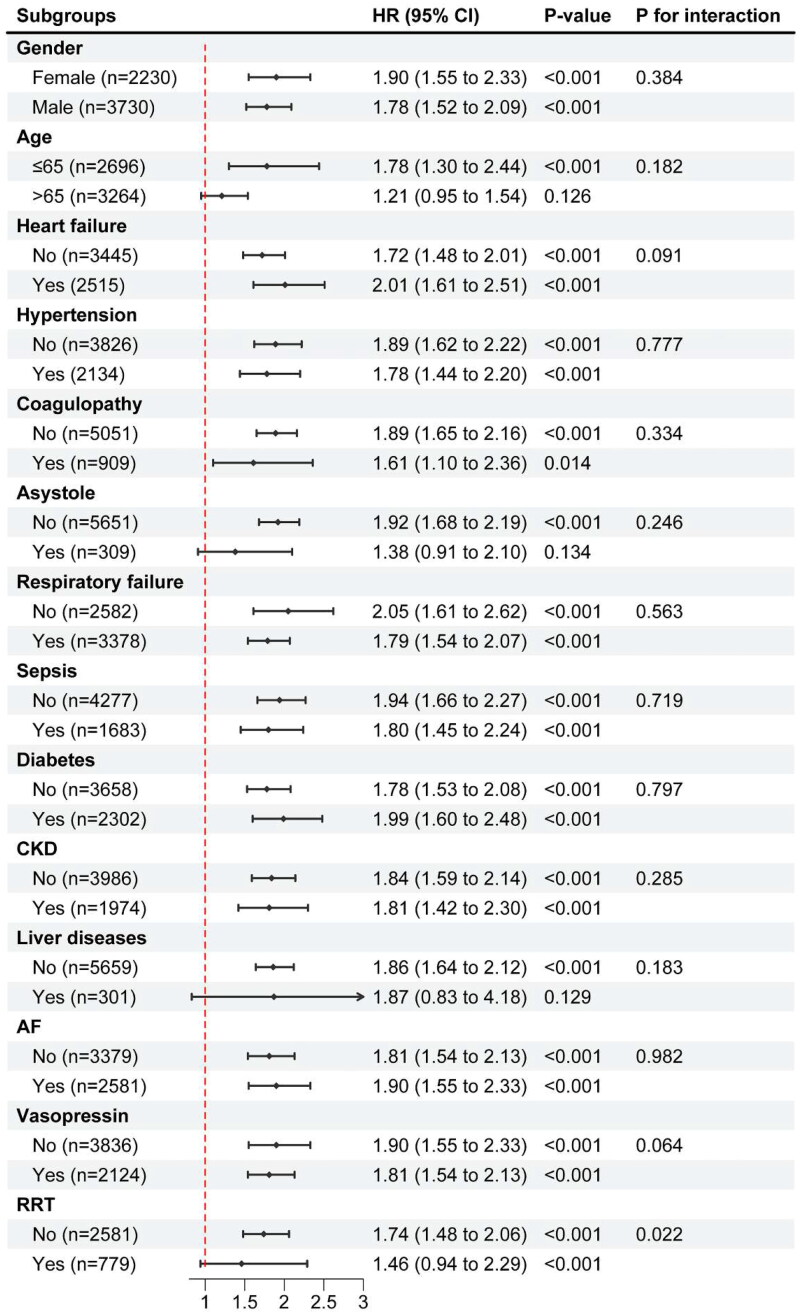
Forest plots for different subgroup analysis of HRs for the association between ePWV and in-hospital mortality.

To confirm the association between ePWV and in-hospital mortality stratified by gender, age, heart failure, hypertension, coagulopathy, asystole, respiratory failure, sepsis, diabetes, CKD, liver diseases, AF, vasopressin, and RRT, subgroup analyses were carried out. As shown in Figure 4, the directionality of effect estimates in all subgroups aligned with the overall outcomes. Moreover, these significant association of increased ePWV and high in-hospital mortality rates mostly affected elderly patients (≤ 65 years), both females and males, individuals with and without heart failure, hypertension, coagulopathy, respiratory failure, sepsis, diabetes, CKD, AF, vasopressin, RRT, and without asystole, liver diseases participants. No significant difference was found among patients with asystole, or liver diseases, possibly because of the limited sample size. Additionally, there was a significant interaction between the subgroup parameters of RRT (*P* for interaction = 0.022).

## Discussion

This study aimed to investigate the association between ePWV and in-hospital mortality in critically ill AKI patients. The results of our study showed that the ePWV was an independent predictor of in-hospital mortality in these patients. After adjusting for confounding factors, higher ePWV remained a significant predictor of in-hospital mortality. Moreover, we found a significant linear correlation between ePWV and the risk of in-hospital mortality in critically ill patients with AKI.

The early detection of AKI is critical for reducing mortality rates. However, detecting AKI using an accurate, simple, and noninvasive marker remains challenging [17]. The commonly used quantifiable markers include serum creatinine, blood urea nitrogen, urinary casts, urine concentration, et al. [18]. It is well known that the usefulness of serum creatinine levels in early AKI detection is limited due to its slow response to changes, which relies on age, sex, and musculature [19]. After hospital discharge, approximately 10–30% of AKI survivors still require dialysis [20]. The outcome of AKI has remained steady in recent years due to a lack of satisfactory biomarkers [21]. Identifying biomarkers for early diagnosis of AKI is still in progress. Since the kidney is a highly vascularized organ, arterial stiffness, which is associated with the risk of cardiovascular mortality, may be one of the mechanisms linking renal insufficiency to cardiovascular events [22,23]. There is evidence suggesting a potential association between the etiology of AKI and ePWV, a measure of arterial stiffness. The patients experience systemic inflammation, leading to impair kidney function, which can contribute to vascular remodeling and increased arterial stiffness [24]. Both increased aortic and brachial-ankle pulse wave velocity (baPWV) have been linked to states of general inflammation in recent studies [25]. Furthermore, renal ischemia and tubular injury in patients may also lead to disturbances in fluid and electrolyte ­balance, including fluid retention and hypertension [26]. Hypertension can influence pulse wave velocity [27]. Neurohumoral activation with heightened activity of the sympathetic nervous system and renin-angiotensin-aldosterone system play a critical role in this scenario [28]. Therefore, an increased vascular tone mediated by neural might constitute a direct pathophysiological link between AKI and increased arterial stiffness. The European standard assessment for arterial stiffness is cfPWV, which requires an experienced operator to measure the pulse waves at two sites [29]. Several devices have been developed and validated for the noninvasive measurement of arterial stiffness using applanation tonometry, piezoelectric mechanotransducers, cuff-based oscillometry, and photodiode sensors [8]. However, using the SphygmoCor CvMS device for PWV measurement easily leads to errors in accuracy owing to incorrect measurement distances by tape [30]. Compared to this simple surface measurement approach, the arteriograph device demonstrated the least variance, but poor agreement with other devices [31]. However, this technique is costly and time-consuming, making it unsuitable for routine clinical practice [32]. Therefore, ePWV calculations based on chronological age, mean blood pressure, their quadratic terms, and interactions have been suggested as they do not require specialized equipment or trained personnel. Moreover, these calculations exhibited comparable performance to true cfPWV measurements in two datasets: the Monitoring Trends and Determinants in Cardiovascular Disease Danish study and the Paris cohort [12]. Researchers consider ePWV a reliable and valid ­surrogate marker of cardiovascular risk based on their ­findings [33].

Recent research has linked elevated arterial stiffness to an increased risk of CKD and AKI following surgery [14,34]. Consistent with these findings, our study demonstrated that high ePWV (≥ 9.65 m/s) are significantly associated with an increased risk of death in patients with AKI. In our results, we observed that the high ePWV group had more prevalent heart failure, or AF. Firstly, we acknowledge that the impact of heart failure, or AF on arterial stiffness measurements. Heart failure can result in fluid overload and congestion, leading to changes in blood volume and pressure, finally resulting in elevated ePWV measurements [35]. Typically, in patients with AF, ePWV values may appear higher compared to individuals with normal sinus rhythm [36]. Then, we performed appropriate adjustments in the cox regression model to minimize the confounding effect of serum potassium, serum sodium, bicarbonate, BUN, Scr, etc. Furthermore, we performed subgroup analyses to compare HRs between patients with heart failure, AF and those without. The results revealed consistency between and no significant differences (Heart failure subgroup: *P* for interaction: 0.091; AF subgroup: *P* for interaction: 0.982), suggesting that heart failure, AF did not significantly affect the reliability of ePWV measurements in our study population.

Vasopressin is known to play a crucial role in cardiovascular regulation and hemodynamic stability [37]. Arterial stiffness is closely linked to various cardiovascular risk factors and diseases such as hypertension, atherosclerosis, and heart failure. These conditions often necessitate the use of vasopressin to manage hemodynamic instability [38]. Subgroup analysis also indicated the in-hospital mortality effect of the ePWV was more pronounced in patients without using vasopressin than in patients using vasopressin. Based on these, vasopressin medical use has a positive effect on survival. In our results, patients with high ePWV exhibited more severe arterial stiffness, and there was a higher prevalence of comorbidities such as heart failure, AF. Therefore, it is expected that the demand for vasopressin would be greater in patients who have high ePWV. Interestingly, we found the high ePWV group has less need for the drug compared with the low group in patients’ baseline characteristics. The reduced requirement for vasopressin in the high ePWV group could be attributed to the following fator: arterial stiffness, as indicated by ePWV, is known to be associated with increased vascular resistance and impaired arterial compliance [39]. In individuals with high ePWV, the arterial system may exhibit reduced responsiveness to vasopressin due to structural changes, potentially resulting in a lower need for the medication to achieve the desired hemodynamic effect [40]. A similar situation occurred in patients receiving RRT treatment. Severe forms of AKI often require renal support through RRT to manage electrolyte imbalances, fluid overload, and other complications [41]. Subgroup analysis also indicated the in-hospital mortality effect of the ePWV was more pronounced in patients without using RRT than in patients using RRT, although the confidence interval for using RRT patients crossed 1. Moreover, there was significant interaction in RRT subgroup (*P* for interaction = 0.022). However, we observed the high ePWV group has less need for RRT in baseline table. We propose the following potential explanations: The optimal timing for initiating RRT in AKI patients remains a topic of debate. While RRT is a vital intervention for severe cases, the timing of initiation may vary based on clinical judgment and the trajectory of kidney function decline [42]. It is possible that some patients in the high ePWV group were managed with a delayed initiation approach, allowing for conservative measures to be implemented first [43]. It is worth further researching the impact of vasopressin or RRT treatment on survival outcomes.

The subgroup analysis also indicated that the better survival was observed in the AKI patients with hypertension, coagulopathy, asystole, respiratory failure, sepsis, or CKD. This outcome might be explained by reverse causality: patients previously diagnosed with these illnesses generally might have been under treatment or might have adopted healthier habits; thus, we observed lower HRs between ePWV and in-hospital mortality [44]. In addition, we should realize that many factors can contribute to mortality in AKI patients in the ICU, in additional to evaluated ePWV. The presence of bleeding in AKI patients can lead to hemodynamic instability, anemia, and compromised organ perfusion [45]. Infection in the setting of AKI can lead to systemic inflammatory response syndrome, sepsis, and septic shock, which significantly impact patient outcomes [46]. Moreover, high ePWV reflects arterial stiffness, which can affect hemostasis and impair immune responses, increasing the risk of bleeding complications and the susceptibility to infections [47]. Regardless, recognizing the important factors contributing to mortality in ICU patients, beyond just ePWV, is crucial. Understanding the interplay between risk factors and ePWV is also vital for comprehending the complex dynamics that influence outcomes in AKI patients.

Despite our promising results, this study had several limitations. First, as all participant data were derived from the MIMIC-IV database, which is a retrospective study. Prospective cohort studies are needed to further detect whether a causal association of high ePWV and mortality exists. Second, our study only included patients with AKI who had been admitted to the ICU, which may exist concerns regarding the hemodynamic status of the patients. Further study needs to determine the relationship between ePWV and AKI in patients who were not hospitalized in the ICU. Third, an inherent limitation of this study is that blood pressure values were acquired before vasoreactive medication administration, which could have mitigated the potential confounding effects. In addition, we unable obtained comprehensive demographic and lifestyle information of patients due to inherent limitations of the Mimic database, Fourth, our analysis did not include an investigation of the cause of death in patients with AKI, particularly cardiovascular mortality. Finally, our investigation focused solely on the predictive value of baseline ePWV in relation to in-hospital mortality in patients with AKI. Future research should explore whether changes in ePWV also predict long-term prognosis. Multicenter, prospective studies are necessary to confirm our findings.

## Conclusion

Our results have expanded the potential applications of ePWV in critically ill patients with AKI, indicating its value as a predictor of hospital mortality in this population. By calculating ePWV, clinicians can significantly aid risk stratification in patients with AKI and gain valuable information about their cardiovascular risk, then develop personalized treatment strategies to improve patient outcomes. Thus, ePWV has the potential to be a simple and readily available biomarker for predicting the risk of death in AKI patients. For future research discoveries, continued efforts to validate ePWV across different populations are crucial to ensure the consistency and comparability of results, enabling broader clinical implementation.

## Data Availability

The data supporting the findings of this study are available from the first author upon reasonable request.
